# Integrated Translatomics with Proteomics to Identify Novel Iron–Transporting Proteins in *Streptococcus pneumoniae*

**DOI:** 10.3389/fmicb.2016.00078

**Published:** 2016-02-03

**Authors:** Xiao-Yan Yang, Ke He, Gaofei Du, Xiaohui Wu, Guangchuang Yu, Yunlong Pan, Gong Zhang, Xuesong Sun, Qing-Yu He

**Affiliations:** ^1^The First Affiliated Hospital of Jinan UniversityGuangzhou, China; ^2^Key Laboratory of Functional Protein Research of Guangdong Higher Education Institutes, Institute of Life and Health Engineering, College of Life Science and Technology, Jinan UniversityGuangzhou, China

**Keywords:** translatomics, proteomics, *S. pneumoniae*, iron-acquisition system, iron-transporting protein

## Abstract

*Streptococcus pneumoniae* (*S.pneumoniae*) is a major human pathogen causing morbidity and mortality worldwide. Efficiently acquiring iron from the environment is critical for *S. pneumoniae* to sustain growth and cause infection. There are only three known iron-uptake systems in *Streptococcal* species responsible for iron acquisition from the host, including ABC transporters PiaABC, PiuABC, and PitABC. Besides, no other iron-transporting system has been suggested. In this work, we employed our newly established translating mRNA analysis integrated with proteomics to evaluate the possible existence of novel iron transporters in the bacterium. We simultaneously deleted the iron-binding protein genes of the three iron-uptake systems to construct a *piaA/piuA/pitA* triple mutant (Tri-Mut) of *S. pneumoniae* D39, in which genes and proteins related to iron transport should be regulated in response to the deletion. With ribosome associated mRNA sequencing-based translatomics focusing on translating mRNA and iTRAQ quantitative proteomics based on the covalent labeling of peptides with tags of varying mass, we indeed observed a large number of genes and proteins representing various coordinated biological pathways with significantly altered expression levels in the Tri-Mut mutant. Highlighted in this observation is the identification of several new potential iron-uptake ABC transporters participating in iron metabolism of *Streptococcus*. In particular, putative protein SPD_1609 in operon 804 was verified to be a novel iron-binding protein with similar function to PitA in *S. pneumoniae*. These data derived from the integrative translatomics and proteomics analyses provided rich information and insightful clues for further investigations on iron-transporting mechanism in bacteria and the interplay between *Streptococcal* iron availability and the biological metabolic pathways.

## Introduction

*Streptococcus pneumoniae* (*S. pneumoniae*) is a Gram-positive bacterium and a major human pathogen. The bacterium is carried asymptomatically in the nasopharynx by up to 70% human population, establishing septicemia, and respiratory tract infections if given the chance to access deeper tissues (Mitchell, 2000; Ong et al., 2013). The capacity of bacterial pathogens such as *S. pneumoniae* to capture iron from the host environment is essential for the establishment of infection (Schaible and Kaufmann, 2004), as the host strictly limits iron sources as a part of its innate defense against invading pathogens. In order to ensure efficient uptake of iron sources, bacterial pathogens have evolved high-affinity iron-uptake ATP-binding cassette (ABC) transporters, with regulated expressions of a range of genes, in response to the limited iron concentration.

ABC transporters share two transmembrane domains (TMDs) that usually form the ligand binding sites with specificity, and two nucleotide-binding domains (NBDs) that bind and hydrolyze ATP to drive the translocation of the bound ligands, transporting a large number of substrates across cellular membranes (Davidson et al., 2008). These ABC transporters are frequently redundant in a strain for efficiently acquiring iron; for example, at least four iron-uptake ABC transporters have been found in *Staphylococcus aureus* (Hammer and Skaar, 2011). In Gram-positive bacteria, substrate-binding proteins (SBPs) of ABC transporters are typical lipoproteins located on cell surface (Hutchings et al., 2009). In *S. pneumoniae*, three ABC transporters, including PiaABC, PiuABC, and PitABC with lipoproteins PiaA, PiuA, and PitA as SBPs, have been identified to be involved in the acquisition of iron, respectively responsible for transporting ferrichrome (Fch), heme, and ferric irons (Brown et al., 2001a, 2002). Recently, two membrane proteins (gi|15901633 and gi|15900732) (Romero-Espejel et al., 2013) and one secreted protein (GAPDH) (Vázquez-Zamorano et al., 2014) have been identified as hemoglobin and haem-binding proteins. Intriguingly, in Brown et al. (2002) study, the *piuB/piaA/pitA* triple mutant poorly grew in iron-restricted medium, but its growth can be restored by adding FeCl_3_ (Brown et al., 2002). We therefore speculated that there are one or more novel iron-uptake systems to sustain the growth of *piuB/piaA/pitA* triple mutant strain. Apart from these, no other novel iron-uptake systems have been suggested to date and the detailed molecular mechanism of iron transportation in *S. pneumoniae* is poorly understood.

To obtain comprehensive information about iron regulation and search for potential iron transporters in *S. pneumoniae*, we used translatomics together with proteomics to assess the global changes in gene and protein expressions in response to the deletions of the three SBP lipoproteins. Translatomics here refers to our newly established translating mRNA analysis, in which mRNAs in the ribosome are purified and sequenced to obtain the comprehensive information on the genes being translated into proteins (Wang et al., 2013b; Zhang et al., 2014). Under steady-states, the abundances of translating mRNAs are highly correlated to the expression levels of proteins on genome-wide scale, and the preferentially translated genes dictate the cellular functions and phenotypes (Wang et al., 2013b; Zhang et al., 2014). With high sequencing coverage and sensitivity, translatome sequencing is capable to detect full-length translating mRNAs corresponding to low abundance proteins, poorly soluble proteins and easily degradable proteins that usually missed by proteomics, and thus becomes an important method complementary to proteomics in identifying protein alterations (Zhang et al., 2014; Zhong et al., 2014).

In this work, we constructed the *piaA/piuA/pitA* triple mutant (Tri-Mut) and investigated the effects of the simultaneous deletion of the three SBP genes on *pneumococcal* growth in various media. We then compared both the translatomes and proteomes in parallel between Tri-Mut and the corresponding wild-type (WT) strain of *S. pneumoniae* D39 using ribosome associated mRNA analysis integrated with iTRAQ-based proteomics. Quantitative results revealed that a large number of genes and proteins are affected by the gene deletions, among which a novel iron-binding protein SPD_1609 belonging to a potential iron-uptake ABC transporter, operon 804, in *S. pneumoniae* was identified and verified.

## Materials and methods

### Ethics approvals

The sheep blood was purchased from Ruite (Guangzhou, China), all procedures were performed in accordance with the China Animal Experimentation and Welfare Ethics Committee.

### Bacterial strains, growth media, and culture conditions

*S. pneumoniae* D39 strain was routinely cultured in Todd-Hewitt broth (Oxiod, UK) containing 0.5% yeast extract (THY) or grew on Columbia agar (Difco, USA) containing 5% sheep blood (Ruite, China) at 37°C with 5% CO_2_. When necessary, appropriate antibiotics were added to media: erythromycin (Erm) at 0.2 μg/mL, chloramphenicol (Cm) at 4 μg/mL, spectinomycin (Spec) at 100 μg/mL, tetracycline (Tet) at 3.5 μg/mL. The iron-restricted medium was produced by adding 5% Chelex-100 (Bio-Rad) to THY for 8 h with continuous agitation, followed by filter sterilization to remove the Chelex-100 and supplementation with 100 μM CaCl_2_ and 2 mM MgCl_2_. The iron content in the medium after Chelex-100 treatment was determined by inductively coupled plasma mass spectrometry (ICP-MS, Thermo Scientific, USA). When required, 20 μM FeCl_3_, hemin, or Fch was added to the iron-restricted medium.

### Construction of the *piaA/piuA/pitA* triple mutant strain (Tri-Mut)

The primers used for this work were listed in Table S1. Tri-Mut was constructed using the previously described method (Wach, 1996; Bayle et al., 2011). Competent cells of WT-D39 strain was transformed with long flanking homology-polymerase chain reaction (LFH-PCR) products, consisting of an antibiotic resistance cassette (Erm, Cm, or Spec) flanked by 500 bp long fragments homologous to the ends of each target gene, *piaA*(*SPD_0915*), *piuA*(*SPD_1652*), or *pitA* (*SPD_0226*). Tri-Mut was made by replacing *piaA, piuA*, and *pitA* genes of WT with gene encoding resistance to erythromycin (Erm), chloramphenicol (Cm), and spectinomycin (Spec), respectively. Transformant was selected with adequate antibiotic-containing Columbia sheep blood agar plates, and confirmed by PCR and Western blotting. The *piaA/piuA* double mutant and *piaA/piuA/1609* triple mutant (Tri-Mut2) were also constructed using a similar method. These mutations were stable after six sequential passages in THY medium without antibiotic selection.

### Ribosome associated mRNAs purification

*S. pneumoniae* WT and Tri-Mut strains were cultured in normal THY medium, and collected at exponential growth phase. WT and Tri-Mut cells were pre-treated with 100 μg/mL chloramphenicol for 20 min and then pelleted by centrifugation (6000 g, 20 min, 4°C), the supernatants were removed, and the pellets were resuspended in 5 mL pre-chilled Buffer B [50 mM Hepes, 500 mM KOAc, 24 mM Mg (OAc)_2_, 100 μg/mL chloramphenicol, pH 7.4] supplemented with 10 mg/mL lysozyme. After 20 min ice-bath, samples were frozen by liquid nitrogen and disrupted using mechanical grinding. Cell lysates were treated with RNase-free DNase I (Thermo Scientific, USA) for 15 min on ice, and the debris was removed by centrifugation at 18,000 rpm for 15 min at 4°C. Supernatants (3 mL) were layered on 12 mL of 35% sucrose buffer, Ribosome associated mRNAs were pelleted after ultracentrifugation (Beckman Coulter SW 70 Ti rotor) at 42,000 rpm for 5 h at 4°C. Ribosome associated mRNAs were isolated using the TRIzol RNA extraction reagent (Ambion, USA) according to the manufacturer's instructions. Genomic DNA was removed by treatment with RNase-free DNase I. The 23S, 16S, and 5S rRNAs were removed using the Ribo-Zero magnetic kit (Gram-Positive Bacteria, Epicentre, USA). The rRNA-depleted mRNA was verified by agarose gel electrophoresis.

### Sequencing and data analysis

The ribosome associated mRNA libraries were generated using NEBNext® mRNA Library Prep Master Mix Set for Illumina (BioLabs, USA) as directed by the manufacturer. The purified libraries were sequenced on an Illumina HiSeq 2000 sequencer for 50 cycles. The reads that passed the Illumina filter were mapped to *S. pneumoniae* D39 reference genome (NC_008533.1) using FANSe2 program (http://bioinformatics.jnu.edu.cn/software/fanse2/) (Xiao et al., 2014) with the following criteria: max read length = 60; max error = 3; indel detection = on; best position = on; min. seed length = 8; memory reduction = on. Differential expression analyses between groups were conducted using edgeR (Robinson et al., 2010). A combined criterion of |log_2_(fold change)|≥ 1 and a *p* < 0.05 was adopted to judge the significance of differentially translated gene (DTG) between WT and Tri-Mut. Two biological replicates were performed.

### Protein preparation, iTRAQ labeling, and proteomics analysis

Proteins were extracted from the WT and Tri-Mut strains cultivated in normal THY medium at exponential growth phase in accordance with our previously reported method (Yang et al., 2015). Two hundred microgram proteins from each sample were dissolved in an equal volume of sample buffer, followed by disulfide reduction with 10 mM of dithiothreitol (56°C, 1 h) and alkylation with 55 mM of iodoacetamide (25°C, 40 min in dark). For each sample, 20 μg proteins were quantified by 10% SDS-PAGE, then 150 μg proteins were precipitated with 4 volume of ice-cold acetone at −20°C for 2 h and collected by centrifugation (2000 g, 5 min, 4°C). The pellet was resuspended in 40 μL dissolution buffer (0.5% TAB, 1 M urea), digested with trypsin (1:25 w/w at 0 h, 1:50 w/w at 3 h) (Promega, USA) at 37°C for 18 h and then lyophilized.

The iTRAQ labeling of the peptide samples were performed using an iTRAQ Reagent 4-plex kit (AB SCIEX, USA) according to the manufacturer's protocol. Two biological replicates for WT were labeled with 114-, 115-, and two biological replicates for Tri-Mut were labeled with 116-, 117-. After incubation for 2 h, the labeled peptides with respective isobaric tags were dried to ~20 μL with a vacuum centrifuge. The labeled WT and Tri-Mut replicate samples were 1:1 pooled (114 vs. 116, 115 vs. 117), and cleaned up using Strata-X 33u polymeric reversed phase column (10 mg/mL, Phenomenex, USA). Desalted peptides were resuspended with buffer A (5% acetonitrile, 0.1% formic acid) and detected using an ABSCIEX Triple-TOF 5600 mass spectrometer (AB SCIEX, USA) coupled with a Nanospray III source and a pulled quartz tip. The parameters were used in the mass spectrometer as described previously (Yin et al., 2013).

The data (.mgf) were acquired from raw data (.wiff) by AB SCIEX MS Data Converter V1.1 software, then identified and quantified via ProteinPilot™ Software 4.5. The quantitative analysis parameters were set as follow: Sample Type, iTRAQ 4 plex (Peptide Labeled); Cys. Alkylation, Iodoacetic acid; Digestion, Trypsin; Instrument, Triple-TOF 5600; ID Focus, Biological modifications; Database, *S. pneumoniae* D39_.fasta.fasta; Search Effort, Thorough; Detected Protein Threshold [Unused ProtScore (Conf)] > 1.30 (95.0%). The criteria of fold chang >1.20 or < 0.83 combined with *p* < 0.05 was used to define the differentially expressed protein (DEP) between WT and Tri-Mut in two biological replicates.

### Functional category and network analysis

The biological processes of the DTGs and DEPs were analyzed according to published data or closely related homologs, combined with (Yu et al., 2012) on the Gene Ontology (GO) database searches using clusterProfiler (v1.12.0) (http://www.bioconductor.org/) of *S. pneumoniae* D39 (Sun et al., 2010). And the interaction networks for DTGs and DEPs were constructed by the STRING as described previously (von Mering et al., 2005, 2007) (http://string-db.org/). The following parameter settings were used: organism *S. pneumoniae* D39; confidence threshold 0.70; no more than 10 interactors. The networks were represented with Cytoscape.

### Immunization experiments and western blotting analysis

Purified His_6_-PiaA, His_6_-PiuA, His_6_-PitA, His_6_-PsaA, and SPD_1609 proteins were used as antigens for the immunization experiments to generate multiclonal antibodies in mice according to the previous report (Brown et al., 2001b). For Western blotting analysis, equal amounts of proteins were separated with 10–20% SDS-PAGE electrophoresis prior to transfer onto polyvinylidenedifluoride (PVDF) membranes (Millipore, USA), then probed with the mouse specific multiclonal antibodies and horseradish peroxidase (HRP)-conjugated goat anti-mouse secondary antibodies. The results were visualized by Clarity™ Western ECL Substrate (Bio-Rad, USA) and quantified using ImageMaster 2D Platinum 6.0. PsaA protein was used as a loading control.

### Analysis of intracellular metal concentration

Bacteria at exponential growth phase (OD_600_ = 0.4~0.5) were pelleted and washed three times with 1 × PBS which pretreated with chelex-100 resin. Subsequently, the cell pellets were dried using a Scanvac Freeze Dryer (Labgene Scientific, Switzerland) and the dry weights were measured. The dry cell mass was resuspended in 65% nitric acid, then heated to 95°C for 20 min. Samples were then diluted to 2% nitric acid and centrifuged at 13,200 g for 30 min, the supernatants were collected and submitted for ICP-MS analysis. Metal contents of samples were normalized to dry weight of cells (ng of Fe per mg cells). All data were evaluated with at least three independent biological experiments.

### Statistical analysis

Statistical analysis was carried out using two tailed, unpaired Student's *t*-test and assigned *p* ≤ 0.05 (denoted by ^*^), *p* ≤ 0.01 (denoted by ^**^) and *p* ≤ 0.001 (denoted by ^***^) by GraphPad Prism version 5.01. Data presented are the mean ± SEM of at least three biological replicates.

## Results and discussion

### The growth of the Tri-Mut was disturbed in iron-depleted media

**Figure 1:**
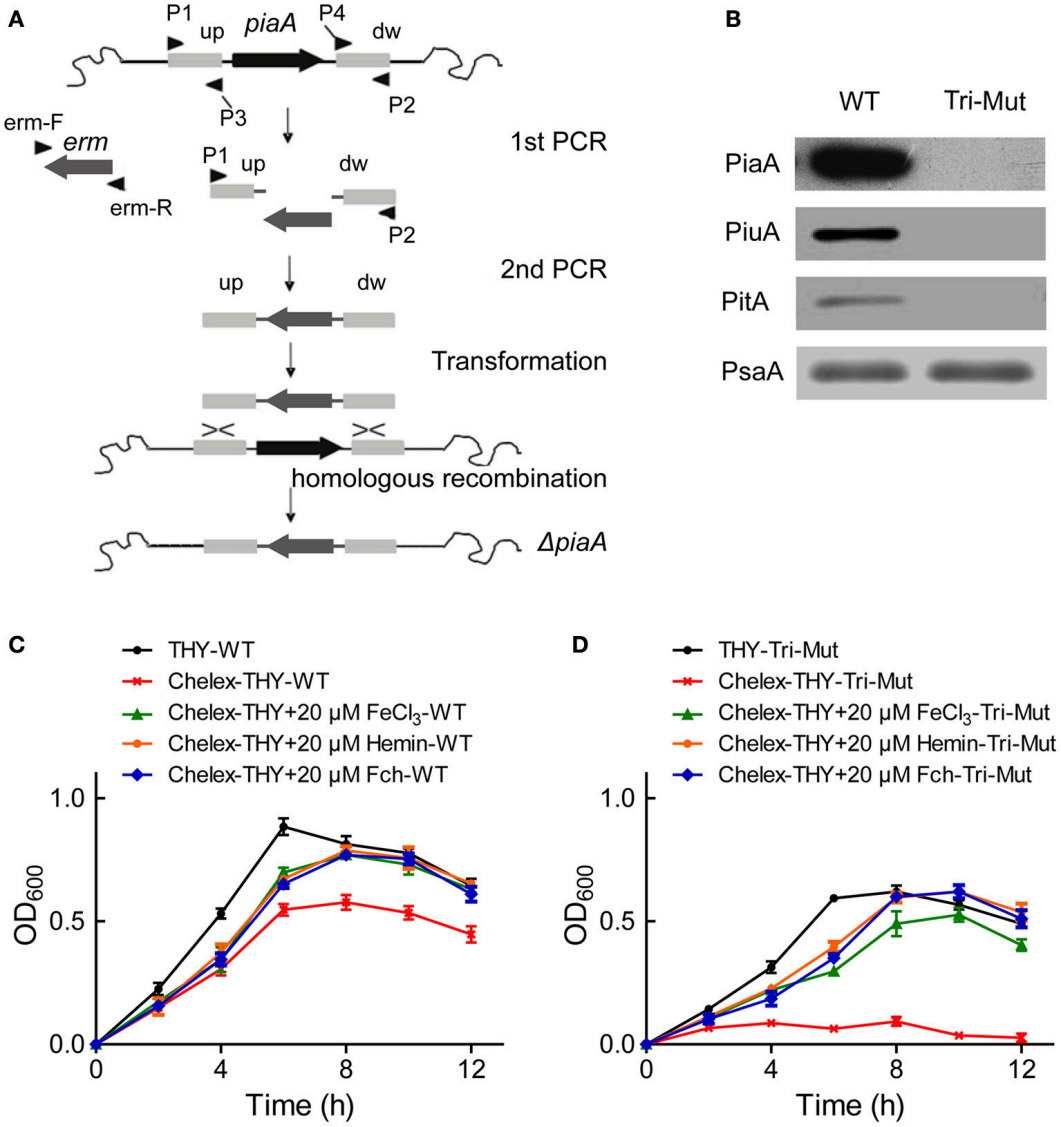
**Construction, verification, and characterization of ***piaA/piuA/pitA*** triple mutant (Tri-Mut) of ***S. pneumoniae*****. **(A)** Strategy map of gene knock-out. **(B)** Verification of the simultaneous deletion of the three genes *piaA/piuA/pitA* in Tri-Mut strain by Western blotting. PsaA protein was used as loading control. **(C)** The growth curves of WT strain in various media with or without iron source. **(D)** The growth curves of Tri-Mut in various media in the presence or absence of iron source.

In *S. pneumoniae*, three operons (*pia, piu, pit*) encode ABC transporters known as iron-uptake systems, each contains one lipoprotein iron receptor (SBP), one ATPase and two transmembrane permease proteins (Brown et al., 2001a, 2002). In order to search for new iron-uptake related proteins, we deleted the three critical SBP genes *piaA/piuA/pitA* in WT-D39 strain using homologous recombination (Figure 1A) to construct the Tri-Mut, with a presumption that genes/proteins related to the iron uptake must be potentially regulated in response to the deletion. PCR (Figure S1) and Western blotting were used to confirm the complete absence of the three SBPs in Tri-Mut (Figure 1B).

We then evaluated the bacterial growth of the Tri-Mut as compared to WT in various media. As shown in Figure 1C, WT strain reasonably had a delayed growth in Chelex-treated iron-restricted medium, and adding any of FeCl_3_, hemin, and Fch can recover the growth. As comparison, Tri-Mut had a decreased growth even in normal THY medium and almost did not grow in iron-restricted medium; interestingly, adding iron sources restored the bacterial growth (Figure 1D). This observation suggests the existence of other iron-acquisition channels that assimilated iron for the bacterial growth in the absence of the three primary SBPs in Tri-Mut. Indeed, potential iron-related genes, and proteins can be stimulated to compensate the defect of the normal iron-uptake systems in Tri-Mut. These genes and proteins were probably up-regulated in Tri-Mut and should be identified by both translatomics and proteomics through the comparison between WT and Tri-Mut.

### Screening for DTGs and DEPs by translatomics and proteomics

**Figure 2:**
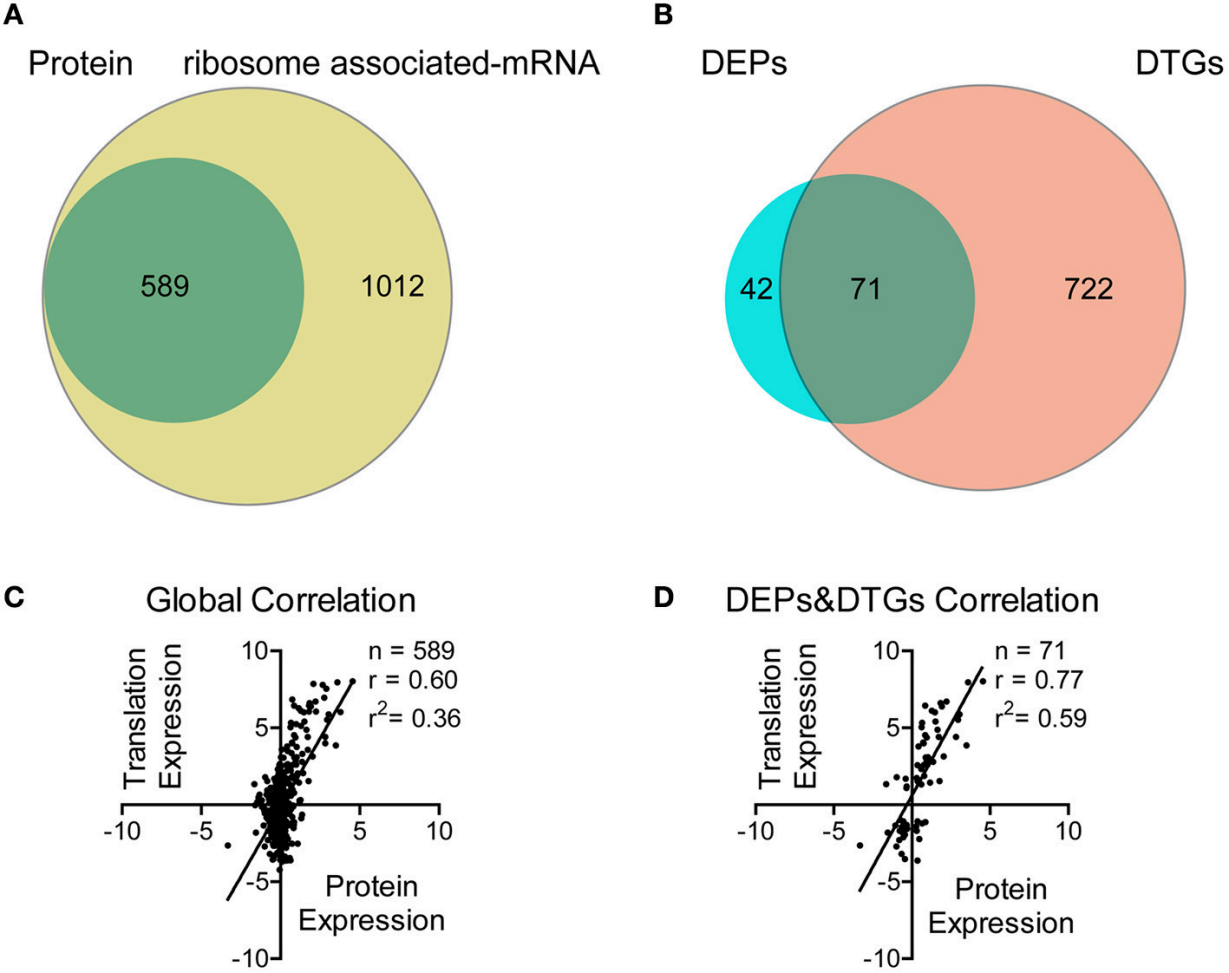
**Correlation analysis between proteomics and translatomics**. **(A)** Venn diagram of the number of proteins and mRNAs quantified using quantitative proteomics and mRNA-Seq, respectively. **(B)** Venn diagram of the number of DEPs and DTGs, respectively. **(C)** Scatterplot of the relationship between the fold changes of proteins and mRNAs (Tri-Mut vs. WT strain, log_2_-transformed) quantified in both data sets. **(D)** Scatterplot of the relationship between the fold changes of DEPs and DTGs (Tri-Mut vs. WT strain, log_2_-transformed) quantified in both data sets.

We firstly used mRNA sequencing to screen for the affected genes in Tri-Mut. A total of 1454 genes in WT and 1666 genes in Tri-Mut were mapped with ≥10 reads, representing ~70.3 and 80.5% of the 2069 predicted genes in the *S. pneumoniae* D39 genome, respectively. The differences in gene expression between WT and Tri-Mut with two biological replicates were calculated by edgeR, resulting in totally 1601 genes (Figure 2A). Using a cutoff of 2.0-fold change and *p* < 0.05, 793 genes were considered as differentially translated genes (DTGs) (Figure 2B), including 430 up-regulated DTGs and 363 down-regulated DTGs in Tri-Mut. More than half of genes in Tri-Mut were regulated as that the simultaneous deletion of three major iron transporter genes in the bacterium seriously destroyed the iron-uptake ability of Tri-Mut and thus affected many important biological pathways.

iTRAQ-based proteomics was then used to identify the proteins with altered expressions in Tri-Mut. Using ProteinPilot™ Software, we detected a total of 589 proteins with isotopic labels for comparison in two biological replicates (Figure 2A). Relative quantitative analysis revealed 113 DEPs with *p* < 0.05, including 52 DEPs with increased abundance (≥1.20 fold) and 61 DEPs with decreased abundance (≤0.83) in Tri-Mut (Figure 2B). The complete list of the DTGs and DEPs is provided in Tables S2, S3 in the Supplemental Materials. The global correlation between translatomics and proteomics data was visualized as scatter-plots (Figure 2C), showing a moderate but significant correlation (*r* = 0.60; *n* = 589, *p* < 0.001). Moreover, the degree of correlation between DEPs and DEGs also exhibited a good correlation (*r* = 0.77; *n* = 71, *p* < 0.001, Figure 2D).

As shown in Figure 2A, the number of genes observed by translatomics were more than those identified by proteomics because ribosome associated mRNA sequencing is independent of physical and chemical properties of proteins. For example, many transmembrane proteins such as SPD_0088, SPD_0089, SPD_1607 were solely detected in mRNA-seq (Table S2), probably due to their low solubility and thus failed to be identified by mass spectrometry. Moreover, we noted that almost all genes of an operon could be found in the translatomics data, but only one or two proteins encoded by the operon genes were identified by proteomics (Tables S2, S3). These results exhibited that full-length translating mRNA analysis possesses higher identification efficiency than proteomics in prokaryotic systems. Indeed, proteins are the end products of genes that carry out biological functions, the genome-wide and bias-free measurements on translational regulation provide a global view for us to assess the networks involved in the iron metabolism, favorable to the identification of new potential iron-binding proteins.

### Integrative analysis of DTGs and DEPs

**Figure 3:**
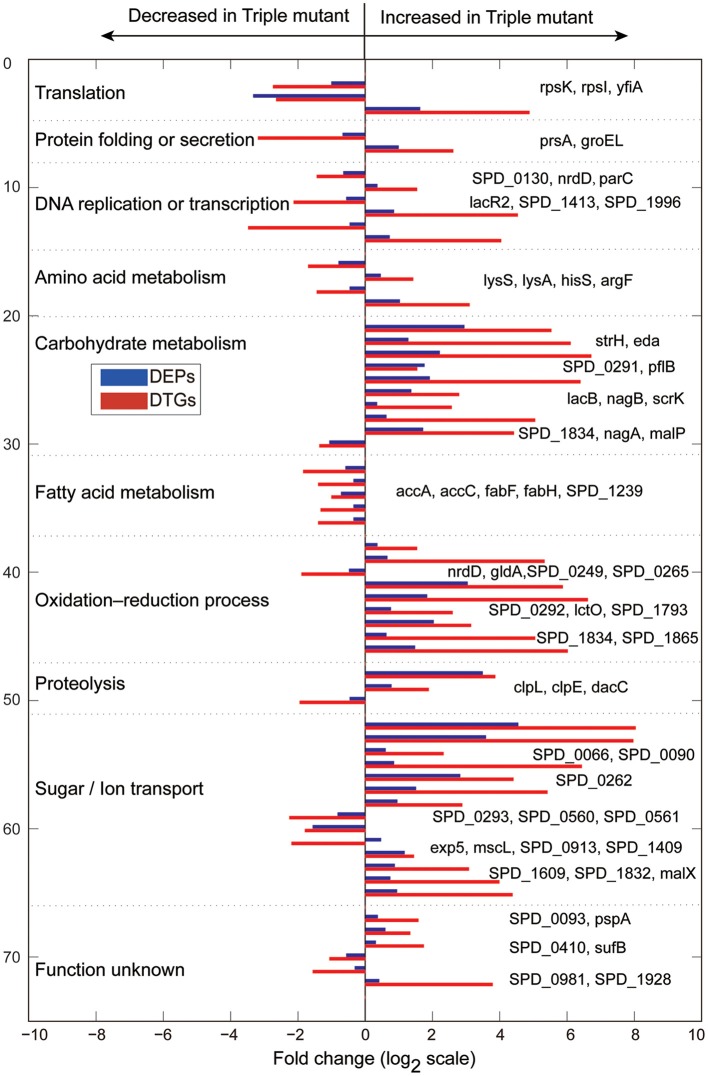
**Biological processes of the DTGs and DEPs between WT and Tri-Mut of ***S. pneumoniae***, which were analyzed according to published data or closely related homologs, combined with the Gene Ontology (GO) database searches using clusterProfiler (v1.12.0) of ***S. pneumoniae*** D39**.

Obviously, the DTGs and DEPs that were simultaneously identified by both translatomics and proteomics are most confidential and promising molecules to be followed up, meaning that they were regulated in both the translating mRNA and protein levels at the same time. By overlapping the two parts of data, we found that 71 DEPs were also identified as DTGs, among which 59 proteins shared the same change trend at both levels; 40 proteins were significantly up-regulated in Tri-Mut while 19 proteins were highly expressed in WT (Figure 2B). Literature searches and related homologs analyses combined with GO analysis were carried out for the biological process of these 59 DEPs and DTGs (Figure 3). These DEPs and DTGs were annotated to various cellular processes including translation, protein folding or secretion, DNA replication or transcription, amino acid metabolism, carbohydrate metabolism, fatty acid metabolism, oxidation–reduction process, and sugar/ion transport (Figure 3 and Table 1).

**Table 1:** **Summary of DEPs and DTGs**.

**Accession**	**Gene name**	**Name**	**DEPs' fold^a^**	***p*-value of DEPs**	**DTGs' fold^b^**	***p*-value of DTGs**	**Operon No**.
**TRANSLATION**							
gi|116515441	rpsK	30S ribosomal protein S11	0.5	0.005	0.15	0.000	102
gi|116517105	rpsI	30S ribosomal protein S9	0.1	0.000	0.16	0.000	133
gi|116516918	yfiA	Ribosomal subunit interface protein	3.09	0.002	29.43	0.000	999
**PROTEIN FOLDING OR SECRETION (CHAPERONE STRESS RESPONSE)**							
gi|116517083	prsA	Foldase protein PrsA	0.63	0.007	0.11	0.000	428
gi|116516636	groEL	Chaperonin GroEL	1.98	0.002	6.11	0.000	842
**DNA REPLICATION OR TRANSCRIPTION**							
gi|116516160	SPD_0130	Metallo-beta-lactamase domain-containing protein	0.64	0.003	0.37	0.000	70
gi|116516887	nrdD	Anaerobic ribonucleoside triphosphate reductase	1.28	0.045	2.9	0.000	101
gi|116515631	parC	DNA topoisomerase IV subunit A	0.68	0.003	0.23	0.000	371
gi|116516466	lacR2	Lactose phosphotransferase system repressor	1.8	0.009	23.13	0.000	522
gi|116516011	SPD_1413	ATP-dependent RNA helicase, putative	0.73	0.048	0.09	0.000	705
gi|116517134	SPD_1996	Fucose operon repressor, putative	1.65	0.025	16.39	0.000	983
**AMINO ACID METABOLISM**							
gi|116517090	lysS	Lysyl-tRNA synthetase	0.58	0.010	0.31	0.000	303
gi|116516303	lysA	Diaminopimelate decarboxylase	1.37	0.011	2.68	0.001	877
gi|116515787	hisS	Histidyl-tRNA synthetase	0.73	0.033	0.37	0.000	960
gi|116516173	argF	Ornithine carbamoyltransferase	2.03	0.009	8.55	0.000	975
**CARBOHYDRATE METABOLISM**							
gi|116517042	strH	Beta-N-acetylhexosaminidase	7.68	0.048	46.14	0.000	28
gi|116515570	eda	Keto-hydroxyglutarate-aldolase/keto-deoxy-phosphogluconate aldolase	2.42	0.000	68.73	0.000	143
gi|116515351	SPD_0291	Hypothetical protein SPD_0291	4.63	0.032	105.06	0.000	143
gi|116517188	pflB	Formate acetyltransferase	3.38	0.003	2.91	0.000	199
gi|116517129	lacB	Galactose-6-phosphate isomerase subunit LacB	3.76	0.039	84.16	0.000	524
gi|116516222	nagB	Glucosamine-6-phosphate isomerase	2.57	0.019	6.89	0.000	614
gi|116516735	scrK	Fructokinase	1.27	0.002	5.93	0.000	769
gi|116515886	SPD_1834	Bifunctional acetaldehyde-CoA/alcohol dehydrogenase	1.54	0.023	33.04	0.000	909
gi|116516233	nagA	N-acetylglucosamine-6-phosphate deacetylase	3.28	0.004	21.33	0.000	919
gi|116515958	malP	Maltodextrin phosphorylase	0.48	0.000	0.39	0.000	956
**FATTY ACID METABOLISM**							
gi|116515640	accA	Acetyl-CoA carboxylase subunit alpha	0.67	0.043	0.28	0.000	181
gi|116515928	accC	Acetyl-CoA carboxylase biotin carboxylase subunit	0.79	0.048	0.38	0.000	181
gi|116516059	fabF	3-oxoacyl-(acyl carrier protein) synthase II	0.61	0.004	0.5	0.009	181
gi|116516000	fabH	3-oxoacyl-(acyl carrier protein) synthase III	0.79	0.010	0.4	0.001	181
gi|116516253	SPD_1239	Acyl-ACP thioesterase, putative	0.79	0.010	0.38	0.000	610
**OXIDATION–REDUCTION PROCESS**							
gi|116516887	nrdD	Anaerobic ribonucleoside triphosphate reductase	1.28	0.045	2.9	0.000	101
gi|116515896	gldA	Glycerol dehydrogenase	1.57	0.043	40.12	0.000	111
gi|116515457	SPD_0249	Hypothetical protein SPD_0249	0.72	0.003	0.27	0.000	118
gi|116516519	SPD_0265	Alcohol dehydrogenase	8.22	0.002	58.39	0.000	127
gi|116516839	SPD_0292	Gluconate 5-dehydrogenase	3.57	0.026	98.08	0.000	143
gi|116517149	lctO	Lactate oxidase	1.69	0.000	6.04	0.000	304
gi|116515416	SPD_1793	Universal stress protein family protein	4.08	0.037	8.83	0.000	887
gi|116515886	SPD_1834	Bifunctional acetaldehyde-CoA/alcohol dehydrogenase	1.54	0.023	33.04	0.000	909
gi|116517097	SPD_1865	Alcohol dehydrogenase, zinc-containing	2.78	0.019	64.61	0.000	918
**PROTEOLYSIS**							
gi|116515968	clpL	ATP-dependent Clp protease, ATP-binding subunit	11.2	0.003	14.56	0.000	149
gi|116516310	clpE	ATP-dependent Clp protease ATP-binding subunit ClpE	1.71	0.025	3.69	0.000	354
gi|116516506	dacC	D-alanyl-D-alanine carboxypeptidase	0.73	0.011	0.26	0.000	378
**SUGAR/ION TRANSPORT**							
gi|116516149	SPD_0066	PTS system, IIB component	23.36	0.006	261.8	0.000	30
gi|116516928	SPD_0090	ABC transporter, substrate-binding protein	11.99	0.017	250.12	0.000	45
gi|116516955	SPD_0262	PTS system, mannose/fructose/sorbose family protein, IID component	1.52	0.035	5	0.000	126
gi|116515558	SPD_0293	PTS system, IIA component	1.8	0.000	86.51	0.000	144
gi|116516148	SPD_0560	PTS system, IIB component, putative	7.05	0.023	21.12	0.000	269
gi|116517184	SPD_0561	PTS system, IIC component, putative	2.84	0.030	42.68	0.000	269
gi|116517154	exp5	PTS system, IIABC components	1.93	0.023	7.38	0.000	324
gi|116516166	mscL	Large conductance mechanosensitive channel protein MscL	0.57	0.005	0.21	0.000	444
gi|116516896	SPD_0913	Hypothetical protein SPD_0913	0.34	0.008	0.29	0.000	454
gi|116515506	SPD_1409	Sugar ABC transporter, ATP-binding protein	2.24	0.007	2.72	0.000	702
gi|116515427	SPD_1609	ABC transporter, substrate-binding protein	1.83	0.027	8.46	0.000	804
gi|116515596	SPD_1832	PTS system, IIB component	1.67	0.042	15.86	0.000	908
gi|116517172	malX	Maltose/maltodextrin ABC transporter, maltose/maltodextrin-binding protein	1.92	0.008	20.76	0.000	957
**UNKNOWN**							
gi|116516874	SPD_0093	Hypothetical protein SPD_0093	1.29	0.004	2.98	0.000	48
gi|116515876	pspA	Pneumococcal surface protein A	1.51	0.011	2.52	0.001	67
gi|116515593	SPD_0410	Hypothetical protein SPD_0410	1.24	0.006	3.33	0.000	193
gi|116515674	sufB	FeS assembly protein SufB	0.68	0.014	0.48	0.003	377
gi|116516210	SPD_0981	Hypothetical protein SPD_0981	0.81	0.003	0.34	0.026	485
gi|116515549	SPD_1928	Hypothetical protein SPD_1928	1.33	0.000	13.77	0.000	952

a *DEPs'Fold represents fold changes of proteins in Tri-Mut vs. WT strain*.

b *DTGs'Fold represents fold changes of genes in Tri-Mut vs. WT strain*.

**Figure 4:**
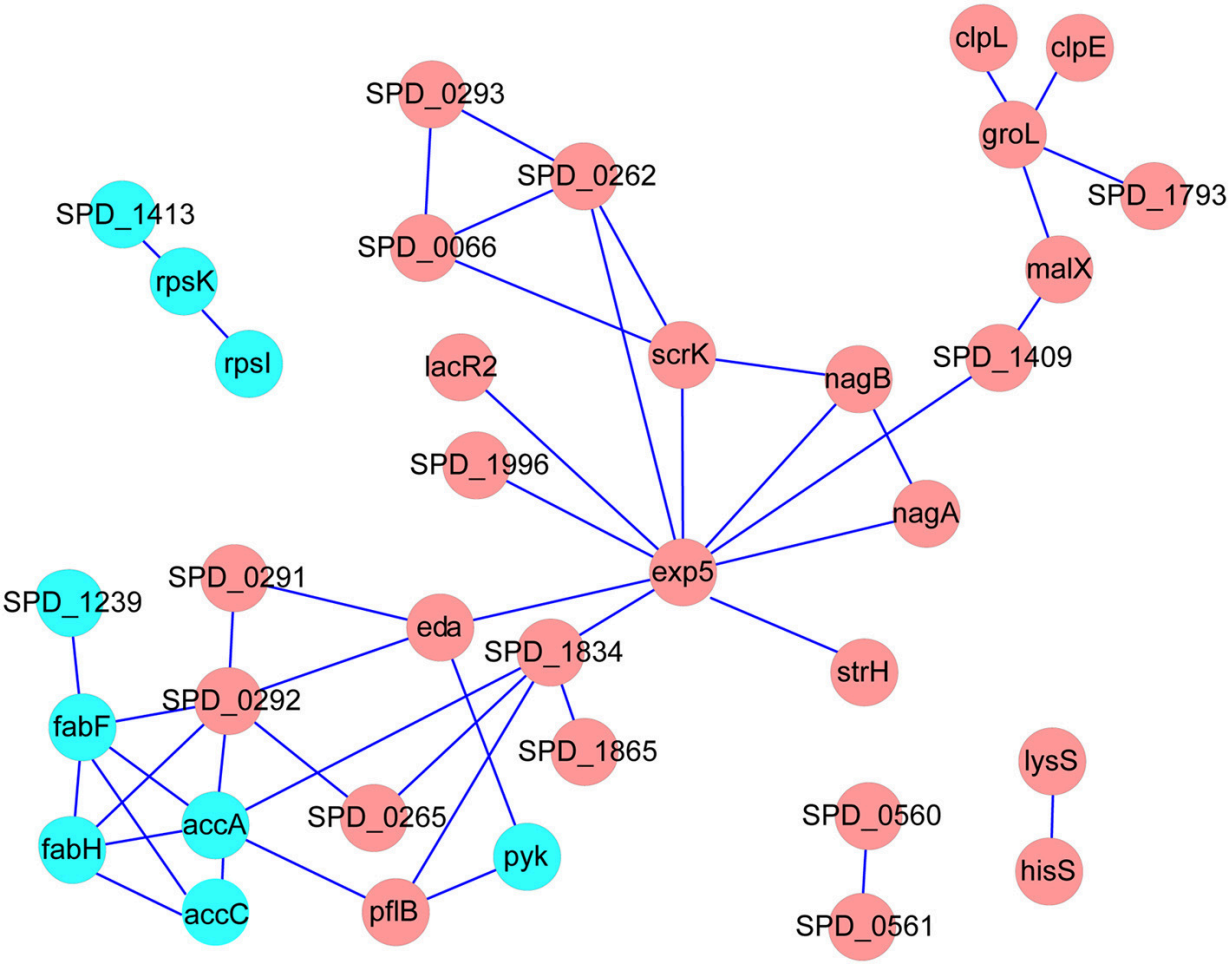
**The interaction networks of DEPs and DTGs were constructed by the STRING database, using confidence level of 0.70, no more than 10 interactors, and the networks were represented with Cytoscape**. Red represents the proteins up-regulated in Tri-Mut, blue represents the proteins down-regulated in Tri-Mut.

We next constructed the interaction networks of the 59 DEPs and DTGs by using STRING database (http://string-db.org/). As shown in Figure 4, most of the DEPs in the map have direct or indirect linkages to each other, forming a large interaction network, suggesting that they are most likely to cooperate in implementing specific biological functions. It is worth noting that five DEPs, including acetyl-CoA carboxylase subunit alpha (AccA), acetyl-CoA carboxylase biotin carboxylase subunit (AccC), 3-oxoacyl-(acyl carrier protein) synthase II (FabF), 3-oxoacyl-(acyl carrier protein) synthase III (FabH), and acyl-ACP thioesterase (putative SPD_1239), involved in fatty acid metabolism as a sub-network, were suppressed in Tri-Mut (Figure 4). Consistently, most genes of this operon within fatty acid metabolism pathway were down-regulated at translational level (Table S2), collectively suggesting that iron is the important cofactor in this process.

In addition, the down-regulation of ribosomal proteins was also detected in Tri-Mut, implicating that iron deficiency inhibited protein biosynthesis, as observed in *Arabidopsis* exposed to iron-deficient conditions (Wang et al., 2013a).

Moreover, seven proteins belong to PTS systems were also induced in Tri-Mut both at mRNA and protein levels (Table 1). The PTS system is traditionally considered as a sugar uptake and phosphorylation system, also mediating catabolite repression in bacteria (Siebold et al., 2001; Lengeler and Jahreis, 2009). Usually, a PTS system is comprised of two cytoplasmic energy-coupling proteins, the Enzyme I (EI) and the histidine-containing protein (HPr), and carbohydrate specific Enzymes II (EII), which is consisted of one or two hydrophobic integral membrane domains (IIC and IID) and two hydrophilic domains (IIA and IIB), responsible for transport- and catalysis-concomitant phosphorylation of the carbohydrate (Deutscher et al., 2006). Previous studies have suggested that PTS system can be considered as the “nerve system” of the bacteria, which employs elaborate signal transduction pathway, responding to external stimuli as well as the internal metabolic status (Aboulwafa and Saier, 2013). The PTS system broadly highly expressed in Tri-Mut, possibly providing carbohydrate to maintain bacterial growth in iron-starved status. This phenomenon had also been found in *Arabidopsis* in responding to iron-deficient stress (Zargar et al., 2015). Nevertheless, whether the PTS system has a side-function involved in iron uptake awaits further investigations.

### Identification of unknown iron-uptake proteins in *S. pneumoniae*

We supposed that potential iron-uptake proteins would be substrate transporters that were highly expressed in the Tri-Mut to acquire iron and maintain bacterial growth, thus we focused on the specifically up-regulated DEPs and DTGs in Tri-Mut. We paid particular attention to the up-regulated DEPs and DTGs assigned to the category of “sugar/ion transport” and “hypothetical protein.” The “sugar/ion transport” category highly expressed in Tri-Mut contains mainly six PTS systems and five ABC transporters.

**Figure 5:**
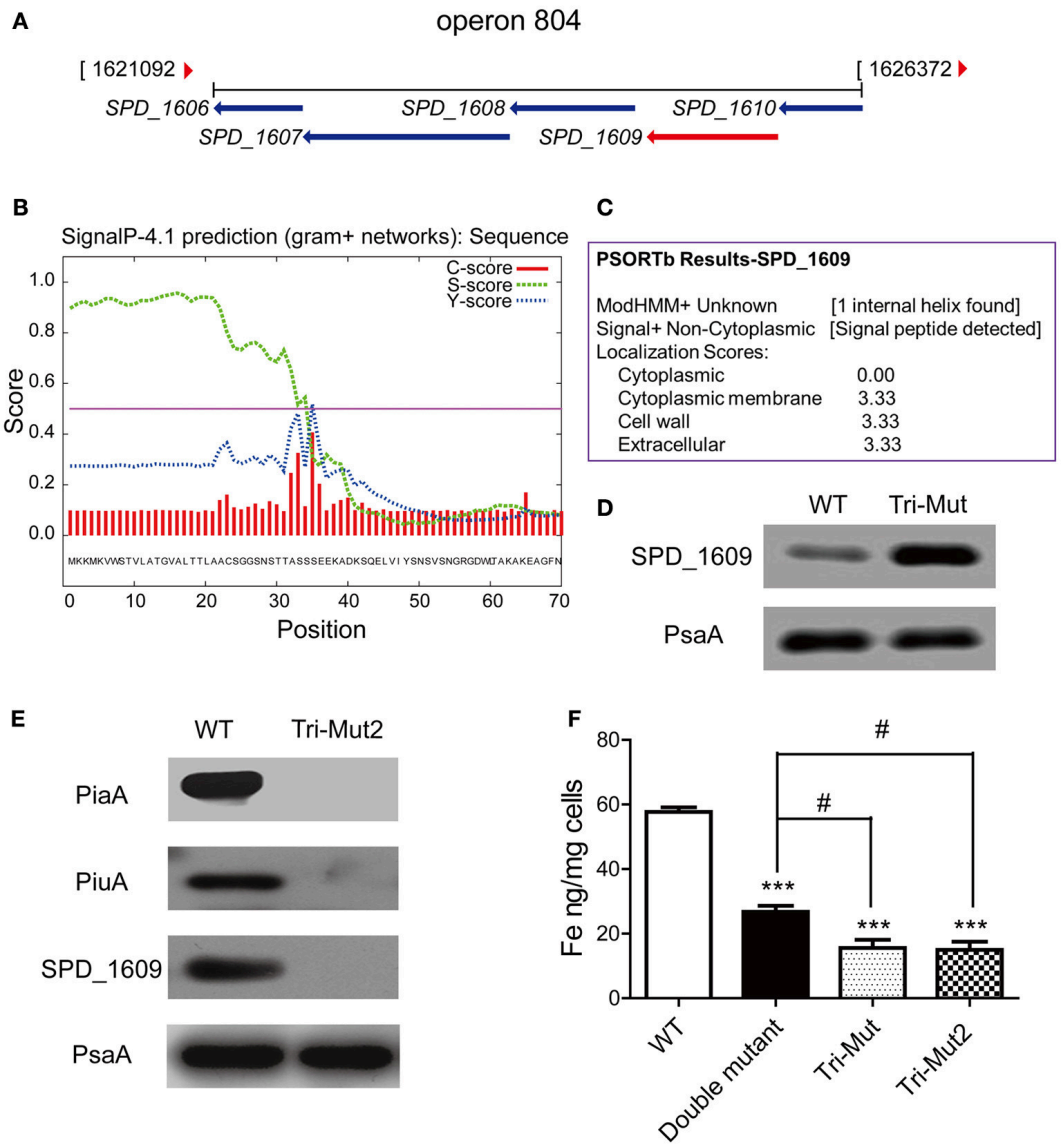
**The operon 804 is probably a novel iron-uptake system required for ***Streptococcal*** growth**. **(A)** Organization of operon 804 in the *S. pneumoniae* D39 genome. **(B)** The SignalP 4.1 analysis on SPD_1609 protein. **(C)** The Psortb analysis on SPD_1609 protein. **(D)** Verification of SPD_1609 protein up-regulation in *piaA/piuA/pitA* Tri-Mut by Western blotting. **(E)** Verification of the complete absence of the three genes *piaA/piuA/1609* in Tri-Mut2 by Western blotting. **(F)** ICP-MS analysis of iron contents in WT strain, *piaA/piuA* double mutant, Tri-Mut, and Tri-Mut2, respectively. Results are the mean of 3 experiments; error bars are SEM, ^***^p < 0.001 vs. WT strain; ^#^*p* < 0.05 vs. *piaA/piuA* double mutant.

In particular, up-regulated ABC transporters here included two sugar ABC transporter proteins, SPD_1409, malX, and two putative conserved ABC transporter SBPs, SPD_0090, and SPD_1609. SPD_0090 protein is located immediately adjacent to two predicted permease proteins SPD_0088 and SPD_0089 of the same transport system (operon 45), while *SPD_1609* gene couples with four neighboring genes: MgtC/SapB family protein (*SPD_1606*), permease protein (*SPD_1607*), ATP-binding protein (*SPD_1608*), and conserved hypothetical protein (*SPD_1610*), forming another ABC transport system (operon 804) (Figure 5A). Supportively, translatomics data demonstrated that all the genes of operons 45 and 804 were up-regulated in Tri-Mut (Table S2).

Further protein sequence alignment revealed that SPD_0090 is highly similar to the SBPs of sugar ABC transporters in many Gram-positive bacteria (Table S4), while SPD_1609 shows a high similarity with the SBPs of iron ABC transporters in *Streptococcus* and *Bacillus* species (Table S5). Moreover, SPD_1609 is a lipoprotein anchored into the cell membrane that contains a typical lipoprotein signal peptide cleavage site (between position 34 and 35 of SPD_1609 protein sequence), which was predicted by SignalP-4.1 (Figure 5B) and Psortb (Figure 5C). These analytical results render SPD_0090 and SPD_1609 appropriate candidates of the SBPs in unknown iron-transport systems, they are potential iron-binding proteins that were up-regulated for iron acquisition compensating to the defect of the primary iron SBPs in Tri-Mut.

### Validation of novel iron-uptake protein SPD_1609 in *S. pneumoniae*

We selected one of the candidates, SPD_1609, for functional validation. We firstly prepared a multiclonal antibody against SPD_1609, and confirmed the up-regulated expression of SPD_1609 in Tri-Mut by Western blotting analysis (Figure 5D). To investigate the biological function of SPD_1609 and exclude the interference of two major iron transporters PiaA and PiuA, we knocked out *SPD_1609* gene in *piaA/piuA* double mutant to generate *piaA/piuA/1609* triple mutant (Tri-Mut2) for the assessment of its biological function. Western blotting experiments shown in Figure 5E confirmed the successful deletion of the three genes *piaA/piuA/1609* at the same time. We also tried several times to construct *pitA/piaA/piuA/1609* tetra mutant, but failed to obtain the mutant strain as it almost did not grow in THY medium. One possible reason is that the simultaneous deletion of the four genes in the bacterium resulted in a complete loss of iron uptake ability. The knock-out of *SPD_1609* gene ensured the complete inactivation of operon 804, the function of the potential iron-transport system can then be evaluated by the comparison among WT, the *piaA/piuA* double mutant, and Tri-Mut2.

We next used ICP-MS to determine the intracellular iron levels in WT, *piaA/piuA* double mutant, Tri-Mut and Tri-Mut2 under identical normal growth conditions. The results are shown in Figure 5F. The deletion of any of the SBP genes in the bacterium would reasonably result in impaired iron acquisition of the mutant strains. As expected, the *piaA/piuA* double mutant presented a substantially reduced intracellular iron concentration as compared to WT. Under this background, deleting one more gene, either *pitA* or *SPD_1609*, further depressed the iron-acquisition ability of the bacterium, leading to the similar decreased levels of intracellular iron in both the triple mutants (Figure 5F). This observation implicates that SPD_1609 may play an iron-binding function similar to PitA; the absence of either the proteins caused an equivalent effect of impairment on iron acquisition. Taken together, these results provided evidences that operon 804 containing *SPD_1609* may function as a novel *Streptococcal* iron-transport system, which works as an important backup for iron metabolism especially when the three known iron-transport systems were inactivated in *S. pneumoniae*.

## Conclusions

In this work, we performed translatomics integrated with proteomics analysis to screen for potential novel iron-transporting proteins in Gram-positive bacteria using *S. pneumoniae* as a model strain. By deleting the genes of three known iron-binding SBPs, a great number of both conventional and previously unreported genes were affected in the Tri-Mut and their corresponding protein alterations were detected by proteomics. With a speculation that potential iron-binding proteins should be up-regulated to compensate the deletion of the three primary SBP genes, we focused on those highly expressed and overlapped DTGs and DEPs in Tri-Mut. Among a number of these up-regulated DEPs, functionally putative proteins SPD_0090 and SPD_1609 were identified to be the potential candidates of novel iron-transporting proteins. Further validations confirmed that SPD_1609 in operon 804 is probably an iron-binding protein similar to PitA. We thus demonstrated a powerful strategy for the search of potential new functional molecules, providing a number of promising candidate genes and proteins related to bacterial iron acquisition. Further validations and investigations on these molecules with putative functions may help to comprehensively understand the iron-transport mechanism, shedding light on the interplay between iron availability and the biological metabolic pathways in bacteria.

The raw data of ribosome associated mRNA sequencing have been uploaded on SRA website (SRP067291). The mass spectrometry proteomics data have been deposited to the ProteomeXchange Consortium (Vizcaíno et al., 2014) *via* the PRIDE partner repository with the dataset identifier PXD003313.

## Author contributions

XY perfomed experiments and wrote paper. KH and GD performed experiments. XW, GY, and YP analyzed data. GZ, XS, and QH instructed experiments and revised paper.

### Conflict of interest statement

The authors declare that the research was conducted in the absence of any commercial or financial relationships that could be construed as a potential conflict of interest.

## Supplementary material

Table S1 **The primer sequences of genes**.

Table S2 **The genes identified by ribosome associated mRNA sequencing-based translatomics**.

Table S3 **The proteins identified by iTRAQ-based proteomics**.

Table S4 **Proteins with high identity and similarity with SPD_0090 based on protein sequence alignment using Position-Specific Iterated BLAST (PSI-BLAST) searches**.

Table S5 **Proteins with high identity and similarity with SPD_1609 based on protein sequence alignment using Position-Specific Iterated BLAST (PSI-BLAST) searches**.

Figure S1 **Verification of ***piaA, piuA, pitA, erm, spec***, and ***cm*** gene by PCR in ***piaA/piuA/pitA*** triple mutant strain**. Lane 1 and 8, DL 2000 DNA Marker; lane 2, *piaA* of WT; lane 3, *piaA* of Tri-Mut; lane 4, *piuA* of WT; lane 5, *piuA* of Tri-Mut; lane 6, *pitA* of WT; lane 7, *pitA* of Tri-Mut; lane 9, *erm* of WT; lane 10, *erm* of Tri-Mut; lane 11, *spec* of WT; lane 12, *spec* of Tri-Mut; lane 13, *cm* of WT; lane 14, *cm* of Tri-Mut.
