# Supplementary material for: Integrated Translatomics with Proteomics to Identify Novel Iron–Transporting Proteins in Streptococcus pneumoniae
Source: Front Microbiol. 2016 Feb 3;7:78. doi: 10.3389/fmicb.2016.00078 (PMC4738293; doi:10.3389/fmicb.2016.00078)
Supplement: Table S1 — The primer sequences of genes. [file Table1.DOCX]

**Table S1 The primer sequences of genes**

| **Primers** | **Sequence (5’ -3’)** |
| --- | --- |
| piaA-P1 | GAAACTCTGAATTATTTGGAAC |
| piaA-P2 | TTTATCTCACCAGTCTTTCCAC |
| piaA-P3 | ATCAAACAAATTTTGGGCCCGG CTCTAACTTGAGCTTTTATGTT |
| piaA-P4 | ATTCTATGAGTCGCTGCCGACT GCCACTTCAAACTCAATTTAAT |
| piaA-F | ACTCAAGCACTAGTCAGACAG |
| piaA-R | TTTGGTACAAGAAAGAGAAAC |
| piuA-P1 | CATTATGGATACAGTTAGC |
| piuA-P2 | TAACTCCAAGACCAACAG |
| piuA-P3 | CTAAATTTTTATCTAAAGTGAATTT GGATCTAGTTTTTATTTTT |
| piuA-P4 | CCTTAACAATCCCAAAACTTGTCGA TGGAATGGGTAATACAAA |
| piuA-F | CTATTTGACGATTTGGATGG |
| piuA-R | TTGAAAGTGGTGTTGGAGTG |
| pitA-P1 | GAATCTCCCAACTCCCCTTT |
| pitA-P2 | GGATTTCTCGGATGGTTTGA |
| pitA-P3 | CATGTATTCACGAACGAAAATCGA CGAACCACCGTCTCCTTTAT |
| pitA-P4 | GAAAACAATAAACCCTTGCATATG CACGATGTCCCTGTTATTGA |
| pitA-F | GAACTAAAAGGAAAAATCGCAA |
| pitA-R | AGCACTAGCAGGTAGGAAGACG |
| 1609-P1 | CTTCGTGGACTGTTTCATCG |
| 1609-P2 | ATGTAAACATAGTGACACCGA |
| 1609-P3 | ATCAAACAAATTTTGGGCCCGG ATTCTTTACATTTCTTGGGC |
| 1609-P4 | TCGTTAAGGGATCAACTTTGGGA AGTAAACTCTTCTTCCTCCTC |
| 1609-F | TTTTGTTCTTTTTGTCCGATT |
| 1609-R | CTACGACTGCTTCTTCATCTG |
| erm-F | CCGGGCCCAAAATTTGTTTGAT |
| erm-R | AGTCGGCAGCGACTCATAGAAT |
| cm-F | AAATT CACTTTAGAT AAAAATTTAG |
| cm-R | TCGACAAGTTTTGGGATTGTTAAGG |
| spec-F | TCGATTTTCGTTCGTGAATACATG |
| spec-R | CATATGCAAGGGTTTATTGTTTTC |
| tet-F | CCGGGCCCAAAATTTGTTTGAT |
| tet-R | TCCCAAAGTTGATCCCTTAACGA |
