# Supplementary material for: Integrated Translatomics with Proteomics to Identify Novel Iron–Transporting Proteins in Streptococcus pneumoniae
Source: Front Microbiol. 2016 Feb 3;7:78. doi: 10.3389/fmicb.2016.00078 (PMC4738293; doi:10.3389/fmicb.2016.00078)
Supplement: Table S4 — Proteins with high identity and similarity with SPD_0090 based on protein sequence alignment using Position-Specific Iterated BLAST (PSI-BLAST) searches. [file Table4.DOCX]

Table S4. Proteins with high identity and similarity with SPD_0090 based on protein sequence alignment using Position-Specific Iterated BLAST (PSI-BLAST) searches.

| Accession No | Function | Organism | Identity | Similarity | Length of amino  acids compared |
| --- | --- | --- | --- | --- | --- |
| WP_000800397 | sugar ABC transporter substrate-binding protein | *Streptococcus oralis* | 473/493  (96%) | 484/493  (98%) | 494 |
| WP_000800426 | sugar ABC transporter substrate-binding protein | *Streptococcus mitis* | 466/493  (95%) | 482/493  (97%) | 494 |
| WP_006149054 | sugar ABC transporter substrate-binding protein | *Streptococcus infantis* | 446/493  (90%) | 471/493  (95%) | 494 |
| WP_012130800 | sugar ABC transporter substrate-binding protein | *Streptococcus gordonii* | 404/491  (82%) | 443/491  (90%) | 486 |
| WP_045758982 | sugar ABC transporter substrate-binding protein | *Streptococcus parasanguinis* | 396/493  (80%) | 441/493  (89%) | 493 |
| WP_044693123 | sugar ABC transporter substrate-binding protein | *Streptococcus suis* | 306/494  (62%) | 376/494  (76%) | 493 |
| WP_034564055 | sugar ABC transporter substrate-binding protein | *Carnobacterium gallinarum* | 272/491  (55%) | 348/491  (70%) | 493 |
| WP_002292274 | ABC transporter substrate-binding protein | *Enterococcus faecium* | 266/486  (55%) | 348/486  (71%) | 486 |
| WP_043031252 | sugar ABC transporter substrate-binding protein | *Streptococcus equi* | 258/491  (53%) | 350/491  (71%) | 481 |
| WP_032461137 | sugar ABC transporter substrate-binding protein | *Streptococcus pyogenes* | 257/491  (52%) | 347/491  (70%) | 481 |
| WP_021037467 | sugar ABC transporter substrate-binding protein | *Lactococcus lactis* | 238/491  (48%) | 326/491  (66%) | 483 |
| WP_028391703 | ABC transporter substrate-binding protein | *Bacillus sp.* FJAT-14515 | 203/492  (41%) | 298/492  (60%) | 485 |
| WP_031644782 | ABC transporter substrate-binding protein | *Listeria monocytogenes* | 179/494  (36%) | 281/494  (56%) | 485 |
