# Supplementary material for: Integrated Translatomics with Proteomics to Identify Novel Iron–Transporting Proteins in Streptococcus pneumoniae
Source: Front Microbiol. 2016 Feb 3;7:78. doi: 10.3389/fmicb.2016.00078 (PMC4738293; doi:10.3389/fmicb.2016.00078)
Supplement: Table S5 — Proteins with high identity and similarity with SPD_1609 based on protein sequence alignment using Position-Specific Iterated BLAST (PSI-BLAST) searches. [file Table5.DOCX]

Table S5. Proteins with high identity and similarity with SPD_1609 based on protein sequence alignment using Position-Specific Iterated BLAST (PSI-BLAST) searches.

| Accession No | Function | Organism | Identity | Similarity | Length of amino  acids compared |
| --- | --- | --- | --- | --- | --- |
| WP_000738379 | iron ABC transporter substrate-binding protein | *Streptococcus mitis* | 338/355  (95%) | 345/355  (97%) | 355 |
| EFO54387 | ABC transporter, substrate-binding protein | *Streptococcus infantis* SK1302 | 294/321  (92%) | 308/321  (95%) | 341 |
| WP_014636980 | iron ABC transporter substrate-binding protein | *Streptococcus suis* | 185/354  (52%) | 253/354  (71%) | 353 |
| WP_000802851 | hypothetical protein | *Bacillus cereus* | 145/349  (42%) | 226/349  (64%) | 355 |
| WP_028389955 | ABC transporter substrate-binding protein | *Bacillus sp.* FJAT-14515 | 133/358  (37%) | 214/358  (59%) | 349 |
| WP_040366038 | iron ABC transporter substrate-binding protein | *Enterococcus italicus* | 87/360  (24%) | 152/360  (42%) | 347 |
| AAK74422  (PitA) | iron ABC transporter, iron-binding protein | *Streptococcus pneumoniae* TIGR4 | 83/340  (24%) | 143/340  (42%) | 338 |
