# Supplementary figures and images for: Integrated Translatomics with Proteomics to Identify Novel Iron–Transporting Proteins in Streptococcus pneumoniae
Source: Front Microbiol. 2016 Feb 3;7:78. doi: 10.3389/fmicb.2016.00078 (PMC4738293; doi:10.3389/fmicb.2016.00078)

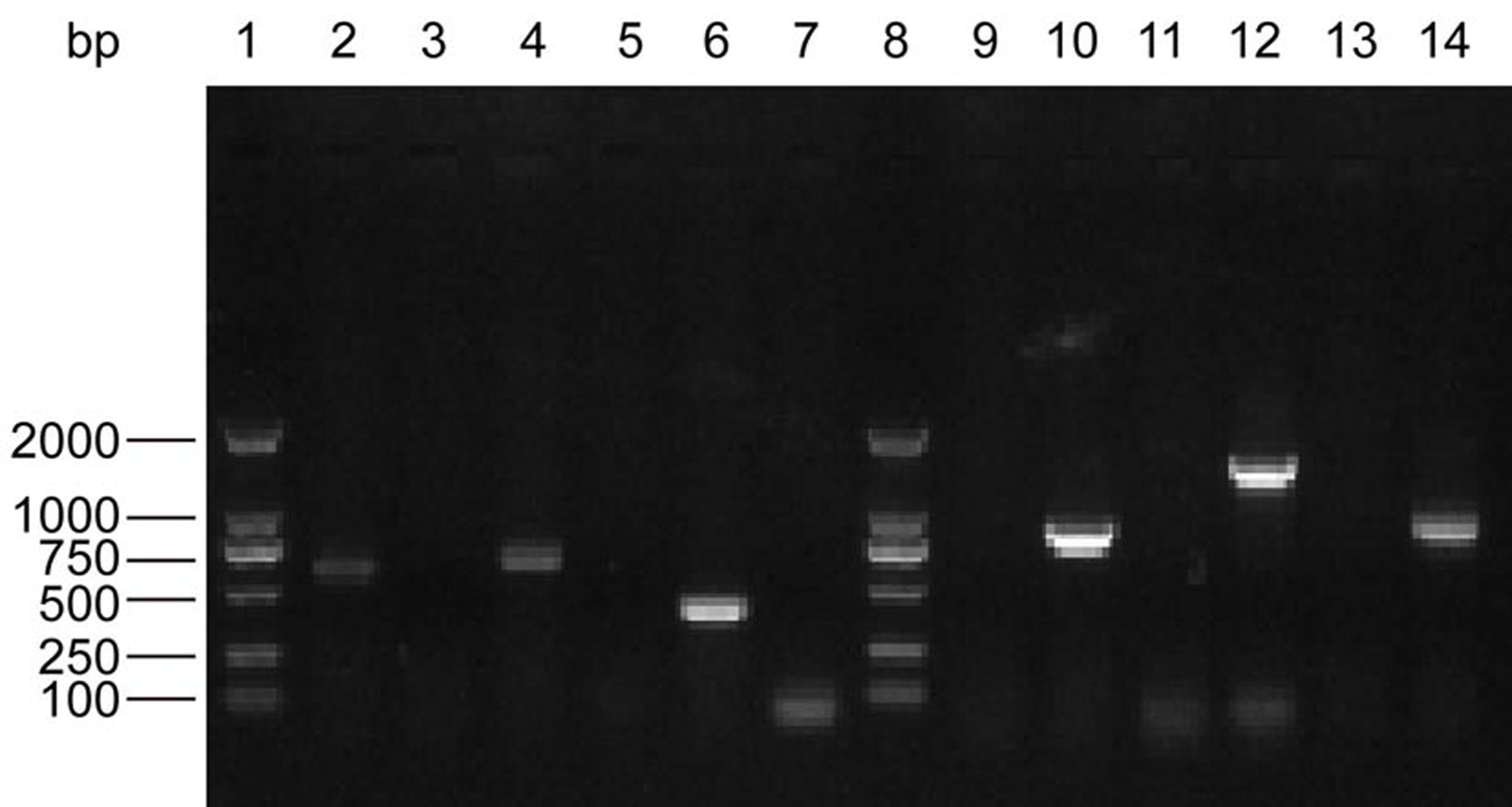

Supplement: Figure S1 — Verification of piaA, piuA, pitA, erm, spec, and cm gene by PCR in piaA/piuA/pitA triple mutant strain. Lane 1 and 8, DL 2000 DNA Marker; lane 2, piaA of WT; lane 3, piaA of Tri-Mut; lane 4, piuA of WT; lane 5, piuA of Tri-Mut; lane 6, pitA of WT; lane 7, pitA of Tri-Mut; lane 9, erm of WT; lane 10, erm of Tri-Mut; lane 11, spec of WT; lane 12, spec of Tri-Mut; lane 13, cm of WT; lane 14, cm of Tri-Mut. [file Image1.TIF]
